# Biosynthesis of Polysaccharides-Capped Selenium Nanoparticles Using *Lactococcus lactis* NZ9000 and Their Antioxidant and Anti-inflammatory Activities

**DOI:** 10.3389/fmicb.2019.01632

**Published:** 2019-07-26

**Authors:** Chunlan Xu, Lei Qiao, Li Ma, Shuqi Yan, Yu Guo, Xina Dou, Baohua Zhang, Alexandra Roman

**Affiliations:** The Key Laboratory for Space Bioscience and Biotechnology, School of Life Sciences, Northwestern Polytechnical University, Xi’an, China

**Keywords:** *Lactococcus lactis*, biogenic, selenium nanoparticles, antioxidant, anti-inflammatory

## Abstract

*Lactococcus lactis* (*L. lactis*) NZ9000, which has been genetically modified, is the most commonly used host strain for nisin regulated gene expression. Selenium (Se) is an essential trace element in the diet of humans and animals important for the maintenance of health and growth. Biosynthesized Se nanoparticles (SeNPs) that use microorganisms as a vehicle are uniquely advantages in terms of low costs, low toxicity and high bioavailability. This study was aimed at preparing novel functionalized SeNPs by *L. lactis* NZ9000 through eco-friendly and economic biotechnology methods. Moreover, its physicochemical characteristics, antioxidant and anti-inflammatory activities were investigated. *L. lactis* NZ9000 synthesized elemental red SeNPs when co-cultivated with sodium selenite under anaerobic conditions. Biosynthesized SeNPs by *L. lactis* NZ9000 were mainly capped with polysaccharides and significantly alleviated the increase of malondialdehyde (MDA) concentration, the decrease of glutathione peroxidase (GPx) and total superoxide dismutase (T-SOD) activity in porcine intestinal epithelial cells (IPEC-J2) challenged by hydrogen peroxide (H_2_O_2_). SeNPs also prevented the H_2_O_2_-caused reduction of transepithelial electrical resistance (TEER) and the increase of FITC-Dextran fluxes across IPEC-J2. Moreover, SeNPs attenuated the increase of reactive oxygen species (ROS), the reduction of adenosine triphosphate (ATP) and the mitochondrial membrane potential (MMP) and maintained intestinal epithelial permeability in IPEC-J2 cells exposed to H_2_O_2_. In addition, SeNPs pretreatment alleviated the cytotoxicity of Enterotoxigenic *Escherichia coli* (ETEC) K88 on IPEC-J2 cells and maintained the intestinal epithelial barrier integrity by up-regulating the expression of Occludin and Claudin-1 and modulating inflammatory cytokines. Biosynthesized SeNPs by *L. lactis* NZ9000 are a promising selenium supplement with antioxidant and anti-inflammatory activities.

## Introduction

Selenium (Se), as an essential trace element, is closely related to human and animal health. Se deficiency is associated with nutritional muscle dystrophy or “white muscle disease” in farm animals (Humann-Ziehank, 2016). Se supplementation can enhance the cellular defense against oxidative stress (Gandin et al., 2018). The synthesis and functions of selenoproteins is dependent on dietary Se (Zoidis et al., 2018). Se exists in nature in two chemical forms including inorganic selenium (selenite, selenate, elemental Se, Se nanoparticles (SeNPs)) and organic Se (selenocysteine, selenomethionine, selenoproteins, etc.) (Biswas et al., 2011; Li et al., 2017). The toxicity order is as follows: selenate > selenite > Se methionine (SeMet) > SeNPs (Nagy et al., 2016; Xu et al., 2018b). In recent years, SeNPs have been widely applied in biomedicine, biosensors, agriculture and environmental remediation due to their unique properties and diverse functions (Nagy et al., 2016; Wadhwani et al., 2016). SeNPs are considered a novel and promising nutritional supplementation (Skalickova et al., 2017).

In recent years, the biosynthesis of SeNPs that employ microorganisms, especially probiotics, has attracted widespread attention and are considered a green process, due to several advantages including safety, cost effectiveness, and an eco-friendly approach and is currently trending as an alternative to traditional methods which include, physical and chemical synthesis methods of SeNPs (Salunke et al., 2014; Shah et al., 2015; Singh et al., 2015; Cui et al., 2016). The probiotic strain *Lactobacillus casei* (*L. casei*) ATCC 393 can effectively transform selenite to SeNPs and accumulates it intracellularly under anaerobic conditions (Xu et al., 2018a,b).

*Lactococcus lactis* (*L. lactis*) as probiotic bacteria are able to express biological proteins or peptides and may also be used as a supplement because of its beneficial effects (Dolatbadi et al., 2015). *L. lactis* NZ9000 exerts a protective effect on dextran sulphate sodium (DSS)-induced colitis in mice (Berlec et al., 2017) and also prevents 5-fluoracil-induced intestinal inflammation in BALB/c mice (Carvalho et al., 2017). The purpose of this study was to develop a benign, cost effective, eco-friendly green process to biologically synthesize SeNPs using *L. lactis* NZ9000 as a vehicle. The physicochemical characteristics of isolated SeNPs were analyzed. Moreover, the antioxidant and anti-inflammatory activities *in vitro* of SeNPs were investigated.

## Materials and Methods

### Cell Line and Reagents

The Porcine jejunal epithelial cell line (IPEC-J2) was originally obtained from the Cell Resource Center, Shanghai Institute of Life Sciences, Chinese Academy of Sciences. All reagents for the cell culture were procured from Life Technologies (Grand Island, NY, USA). Interleukin-6 (IL-6), interleukin-8 (IL-8), tumor necrosis factor-α (TNF-α), and interferon-γ (IFN-γ) Assay Kits were purchased from R&D Systems (Minneapolis, MN, USA). Total superoxidase (T-SOD), glutathione peroxidases (GPx) and malondialdehyde (MDA) Assay Kits were obtained from Nanjing Jiancheng Bioengineering Institute (Jiangsu, China). The Occludin, Claudin-1 and β-actin primary antibodies and secondary HRP antibodies were purchased from Abcam Biotechnology (Cambridge, MA, USA). The Bicinchoninic acid (BCA) protein assay Kit and RIPA lysis buffer were procured from Solarbio Life Sciences Co. (Beijing, China). The Hoechst 33342 Staining Kit, adenosine triphosphate (ATP) Assay Kit, mitochondrial membrane potential (MMP) Assay Kit, reactive oxygen species (ROS) Assay Kit and the Cell Counting Kit-8 (CCK-8) were procured from Beyotime Biotechnology (Shanghai, China). Sodium selenite and fluorescein isothiocyanate-dextran (FITC-dextran, 4 kDa) was purchased from Sigma-Aldrich (St. Louis, MO, USA).

### Bacterial Strains and Culture Medium

Enterotoxigenic *Escherichia coli* K88 (ETEC K88) was kept in our laboratory. *L. lactis* NZ9000 (MoBiTech, Goettingen, Germany) was used for biogenic synthesis of SeNPs. Luria-Bertani (LB) broth was purchased from Oxoid (Basingstoke, England). The M17 broth was purchased from Difco (Detroit, MI, USA).

### Preparation of SeNPs by *L. lactis* NZ9000

First, *L. lactis* NZ9000 was grown in M17 broth (M17B) containing 0.5% glucose at 30°C without shaking. 0.6 mM of sodium selenite was then added to the culture and further incubated for 48 h at 30°C when the optical density at 600 nm of the culture medium was about 0.4. The color change of the medium and bacteria was observed throughout the entire cultivation process. After coculture, fermentation broth was collected and centrifuged at 10,000 rpm for 15 min at 4°C. Bacteria were washed twice with a phosphate-buffered saline (PBS) of pH 7.4. Partial fermentation broth was filtered with a 0.22 μm filter. The colors of the membrane and filtered liquid were also observed. Second, SeNPs were isolated by the methods established in our laboratory (Xu et al., 2018b).

### Composition Analysis of Isolated SeNPs

Se content in isolated SeNPs was detected by Flame emission atomic absorption spectrometer (ZEEnit700, Analytik Jena, Germany). The protein concentration was measured by a BCA Kit according to the manufacturer’s instructions. The polysaccharide concentration of extracted SeNPs was determined using a phenol-sulfuric acid method.

### Characteristics of Isolated SeNPs

Transmission electron microscope (TEM) of Model JEM-1230 (JEOL, Tokyo, Japan) at 80 kV was used to visualize the location and size of SeNPs accumulated in *L. lactis* NZ9000 under standard operating conditions. The particle shape and size of extracted SeNPs were analyzed using scanning electron microscopy (SEM) (Philips, Netherlands) at 3.0 kV. The elemental composition of SeNPs was analyzed by Energy dispersive X-ray (EDX) equipped with EDAX XL-30 system and X-ray photoelectron spectroscopy. In addition, the functional groups of SeNPs were measured by Fourier Transform Infrared Spectrometer (FTIR) (PerkinElmer, Spectrum GX, USA) in the frequency range of 4,000–400 cm^−1^.

### Antioxidant Activity and Cytotoxicity Analysis of SeNPs

First, 1 × 10^5^ IPEC-J2 cells were seeded in 6-well plates (Corning, NY, USA) and cultivated for 12 h. Then IPEC-J2 cells were administrated with 500 μM H_2_O_2_ and (or) SeNPs containing 6 μg/ml Se or fetal bovine serum (FBS)-free DMEM/Ham’s F-12 (1:1) medium for a further 12 h. The morphology of cells was observed under an optical microscope. Moreover, living cells were visualized and counted under an inverted fluorescence microscope (Leica DMIL, Germany) with 6 μg/ml Hoechst 33342 staining at 37°C for 10 min. MDA content, T-SOD and GPx activities were measured with the colorimetric method using the corresponding assay kits.

The cellular toxicity of different selenium species was evaluated in IPEC-J2 cells. Briefly, IPEC-J2 cells were seeded into 96-well plates and cultivated until the confluence rate reached to 90%. Then cells were exposed to sodium selenium, selenium methionine, or SeNPs containing 0–64 μg/ml Se for 12 h, respectively. After treatment, cells from each well were incubated with 10 μl CCK-8 for 2 h. The optical density was determined by a microplate reader (Bio-Rad, Hercules, CA, USA) at 570 nm.

### Effect of SeNPs on Intestinal Epithelial Permeability

IPEC-J2 cells (1 × 10^5^/well) were grown on porous upper inserts of 24-well Transwell plates (Corning, NY, USA). The cell treatment method was the same as detailed above. Transendothelial electrical resistance (TEER) was detected by a Millicell Electrical Resistance System (Millipore, Billerica, MA, USA). After the TEER was detected, 100 μl FITC-Dextran at the concentration of 2.2 μg/ml was added to the upper chamber of the transwells. After being continuously cultivated for 2 h, 100 μl culture medium were collected from the upper and lower sides of the chamber, respectively. FITC-Dextran concentration was determined by Multimode Microplate Reader (BioTeck, USA) at excitation and emission wavelengths of 490 nm and 520 nm, respectively.

### Intracellular Adenosine Triphosphate Levels

IPEC-J2 cells were grown on 6-well plates at a density of 2 × 10^5^ cells/well overnight. As for the SeNPs protective groups, cells were treated with SeNPs at the concentration of 6 μg Se/ml. The Control and H_2_O_2_-model groups cells were given an equal volume of FBS-free medium. After incubation for 12 h, cells from H_2_O_2_-induced oxidative damage groups were treated with 500 μm H_2_O_2_ for 6 h. Finally, all experimental cells were collected and lysed. Intracellular ATP levels were detected by ATP Assay Kit.

### Effect of SeNPs on Reactive Oxygen Species Production

The grouping and treatment of experimental cells were the same as above. The effect of SeNPs and (or) H_2_O_2_ on intracellular ROS production was evaluated by a ROS Assay Kit with 2′, 7′-dichlorodihydrofluorescein diacetate (DCFH-DA) staining according to the manufacturer’s instructions.

### Effect of SeNPs on Mitochondrial Membrane Potential

The grouping and treatment of experimental cells were the same as above. After treatment, MMP was measured by MMP Assay Kit. Briefly, cells from each experimental group were stained with JC-1 for 20 min, and then washed three times with JC-1 buffer. Finally, green monomeric and red aggregated fluorescence images were captured on a Laser Scanning Confocal Microscope (Leica sp5, Germany) at excitation with emission wavelengths of 530 nm and 590 nm, respectively.

### Antagonistic Effect of SeNPs on the Cytotoxicity on IPEC-J2 Cells Caused by Enterotoxigenic *Escherichia coli* K88

ETEC K88 was cultivated in LB broth with shaking at 120 rpm at 37°C overnight. ETEC K88 culture supernatant were collected by centrifuging the 1 × 10^8^ CFU/ml ETEC K88 broth at 5,000 × g for 10 min and used for the following experiments. 1 × 10^5^ cells IPEC-J2 cells were seeded into 6-well plates for 24 h to allow cell attachment. The infective groups were cultivated with an ETEC K88 culture supernatant for 2 h after treatment with SeNPs for 3 h. The morphology of cells was observed under an optical microscope. Living cells were visualized under an inverted fluorescence microscope with Hoechst 33342 staining.

### Regulatory Effect of SeNPs on the Expression of Tight Junction Proteins

The IPEC-J2 cells treatment method was the same as described above. After treatment, cells were washed twice with PBS. Total protein was extracted by dissolving cells in 500 μl RIPA lysis buffer. Protein concentration was measured by BCA Protein Assay Kit. 40 μg protein from each group was boiled with 5X loading buffer at 95°C for 5 min and then loaded to 10% sodium dodecyl sulfate polyacrylamide gel electrophoresis (SDS-PAGE). Proteins were then transferred to a polyvinylidene difluoride (PVDF) membrane (Millipore, Massachusetts, USA). After being blocked with 5% skim milk blocking buffer for 2 h, the PVDF membranes with proteins were incubated with antibodies for Occludin, Claudin-1 and β-actin at 4°C overnight. Subsequently, the membranes were washed three times with Tris buffered saline with Tween 20 (TBST), and then incubated with secondary antibodies for 2 h. After the membranes were washed three times with TBST, the immunoreactive bands were detected and visualized using a Clarity Western ECL substrate Kit (BioRad, CA, United States). The gray values of the bands were quantified by the built-in software.

### Effect of SeNPs on Cytokines in IPEC-J2 Cells Challenged by Enterotoxigenic *Escherichia coli* K88

The IPEC-J2 cells treatment method was the same as described above. After treatment, cell culture medium was collected into 1.5 ml tubes. The levels of IL-6, IL-8, IFN-γ and TNF-α in the cell culture medium were measured by corresponding ELISA assay Kits.

### Statistical Analysis

Data are presented as the mean ± standard error of mean (S.E.M.) in at least three independent experiments and analyzed by one-way analysis of variance (ANOVA) and Student’s *t* test (SPSS 19.0, Chicago, IL, USA). *p* < 0.05 was considered statistically significant.

## Results

### Effect of Sodium Selenite on the Growth of *L. lactis* NZ9000 and Selenite Percent Conversion

**Figure 1:**
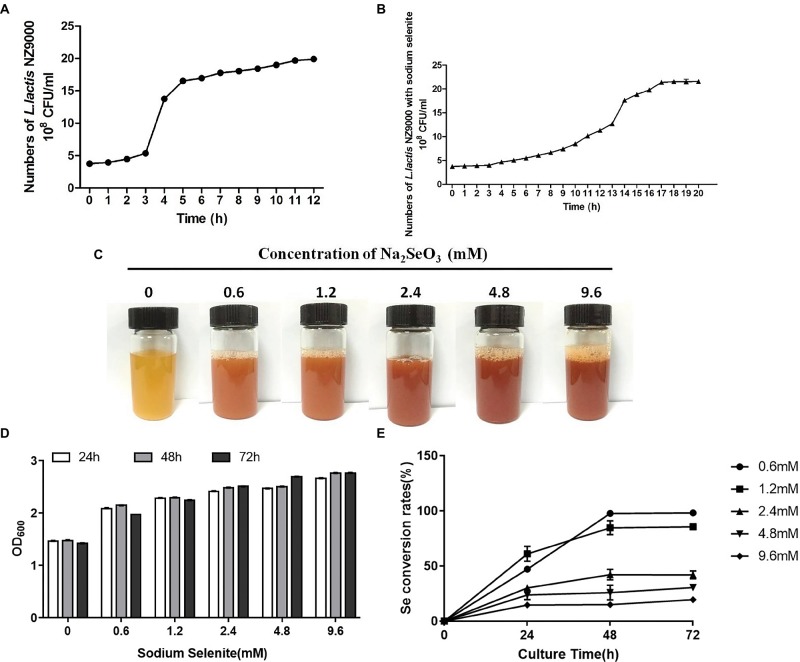
Effect of sodium selenite with different concentrations on the growth of *L. lactis* NZ9000 and selenite percent conversion. **(A)** The growth curve of *L. lactis* NZ9000. The growth of *L. lactis* NZ9000 entered the stationary phase when it was cultivated for 6 h. **(B)** The growth curve of *L. lactis* NZ9000 cultivated with sodium selenite. The growth of *L. lactis* NZ9000 entered the stationary phase at 17 h. **(C)** Apparent color of *L. lactis* NZ9000 culture medium when cultivated with a different concentration of sodium selenite. **(D)** Effect of sodium selenite with different concentration on the growth of *L. lactis* NZ9000. **(E)** The selenite percent conversion when *L. lactis* NZ9000 was cultivated with a different concentration of sodium selenite.

*L. lactis* NZ9000 entered the stationary phase when cultivated for 6 h. However, when cultivated with sodium selenite, *L. lactis* NZ9000 entered the stationary phase for 17 h (Figures 1A,B). Moreover, with the increase of selenium concentration, the color of the culture medium gradually deepened (Figure 1C). Different stress times with the same concentration of sodium selenite did not affect the growth of *L. lactis* NZ9000 (Figure 1D). The selenite percent conversion was close to 100% when *L. lactis* NZ9000 was cultivated with 0.6 mm of sodium selenite for 48 h at 30°C (Figure 1E).

### Biosynthesis of SeNPs by*L. lactis* NZ9000

**Figure 2:**
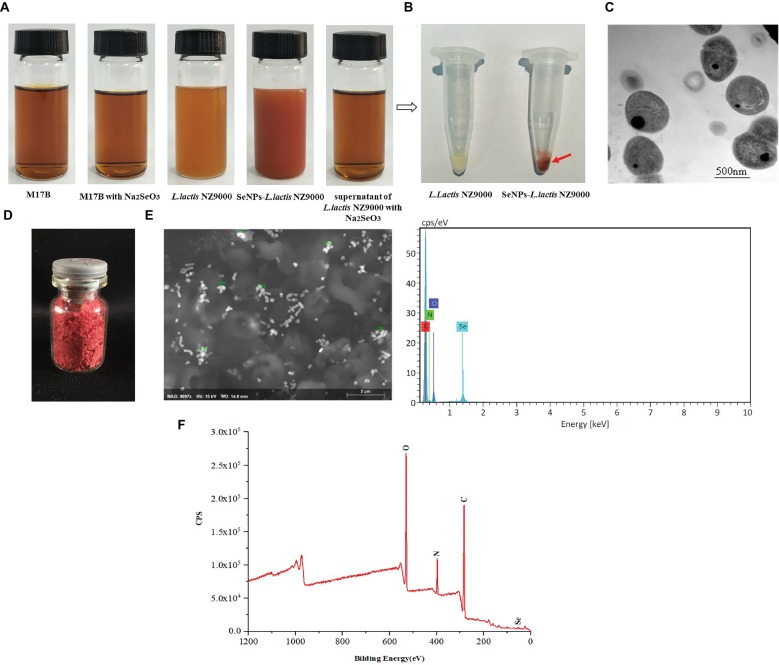
Preparation and characterization of biosynthesized SeNPs by *L. lactis* NZ9000. **(A)** Appearance color of M17B, *L. lactis* NZ9000 supernatant or *L. lactis* NZ9000 cultivated with or without Na_2_SeO_3_ at 30°C for 48 h; **(B)** Visible white color of *L. lactis* NZ9000. **(C)** TEM images of SeNPs-enriched *L. lactis* NZ9000. **(D)** The freeze-dried powder of SeNPs isolated from SeNPs-enriched *L. lactis* NZ9000 appears distinct bright red color. **(E)** SEM–EDX images of isolated SeNPs. **(F)** Extracted biogenic biomolecules capped-SeNPs contained C, N, O and Se elements by X-ray photoelectron spectroscopy (XPS) analysis. SeNPs, selenium nanoparticles.

In order to promote the biosynthesis of SeNPs by *L. lactis* NZ9000, the *L. lactis* NZ9000 strain was cultivated with sodium selenium at the concentration of 0.6 mm under anaerobic conditions. In the whole process of cultivation, we found that M17B culture medium and *L. lactis* NZ9000 supernatant with or without sodium selenite all present transparent deep yellow. However, *L. lactis* NZ9000 after being cultivated with sodium selenite for 48 h, exhibited a distinct bright deep-red color (Figure 2A). After centrifugation, *L. lactis* NZ9000 and SeNPs-enriched *L. lactis* NZ9000 presented white and red color, respectively (Figure 2B). TEM images showed that SeNPs distributed within *L. lactis* NZ9000 with the size range of 38–152 nm (Figure 2C).

### Chemical Composition of Isolated SeNPs

The chemical composition of isolated SeNPs is shown in Table 1. Se, polysaccharides and protein accounted for 6.67, 18.51 and 74.82%, respectively, which indicated that these isolated SeNPs were capped by polysaccharides and proteins.

**Table 1:** Chemical composition analysis.

Constituents	Concentration (%)
Se	6.67
Protein	18.51
Polysaccharide	74.82

### Characteristics of Isolated SeNPs

**Figure 3:**
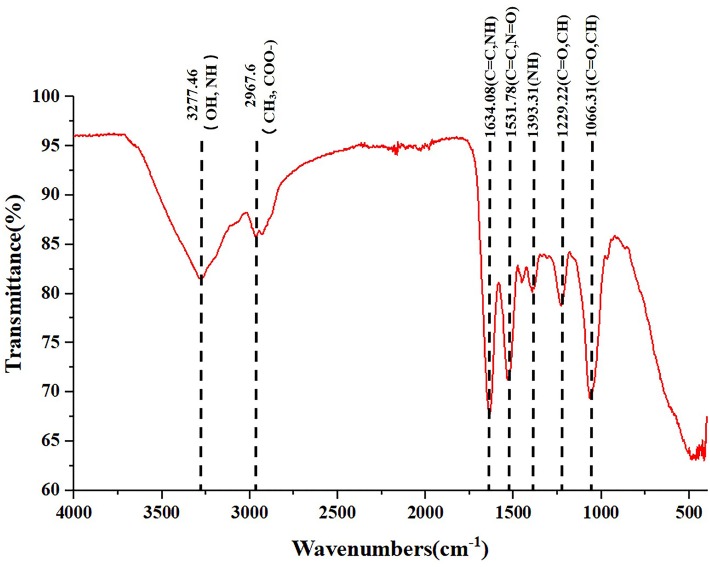
FT-IR spectrum of polysaccharide and proteins-capped biogenic SeNPs synthesized by *L. lactis* NZ9000. FT-IR, Fourier transform infrared spectroscopy.

The freeze-dried powder of isolated SeNPs also present a red color (Figure 2D).As shown in Figure 2F, a carbon (C), oxygen (O), nitrogen (N), and a Se atom signal appeared in the EDX spectrum. SEM images showed that isolated SeNPs present homogeneous particles of 143 nm (Figure 2E). Moreover, the signals of C, N, O and Se were also detected in the XPS spectra (Figure 2G). Furthermore, the band at 3277 cm^−1^ which corresponds to the stretching vibration peak of O-H and N-H was recorded in the FT-IR spectroscopy. The band at 2968 cm^−1^ was attributed to the stretching vibration of C-H. The band at 1634 cm^−1^ depicted the stretching vibration of C=C, C=N. The band at 1229 and 1,066 cm^−1^ represents the stretching vibration of C=O (Figure 3). These data further demonstrated that isolated SeNPs were capped by polysaccharides and proteins.

### Antioxidant Activity and Cytotoxicity of SeNPs

**Figure 4:**
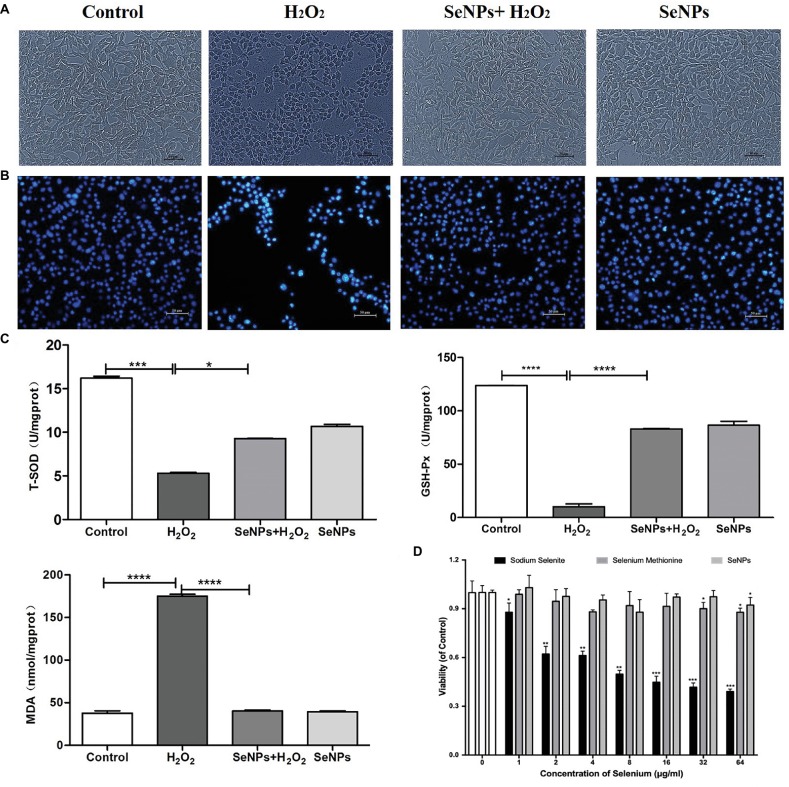
Protective effect of biosynthesized SeNPs by *L. lactis* NZ9000 on H_2_O_2_-induced oxidative damage of porcine intestinal epithelial cells. **(A)** Antagonistic effect of biogenic SeNPs synthesized by *L. lactis* NZ9000 on H_2_O_2_-induced oxidative damage of IPEC-J2 cells. **(B)** Living cells were visualized by fluorescence microscope with Hoechst 33342 staining. **(C)** MAD levels, T-SOD and GPx activity in cultural medium. **(D)** Cytotoxicity of different chemical form of Se on IPEC-J2. Cell viability was quantified by CCK-8 assay. Results were verified by three repetitions of experiments. Different forms of the Se treatment groups vs. the corresponding control. All data were presented as mean ± S.E.M. of three separate experiments. ^*^p < 0.05; ^***^p < 0.001; ^****^p < 0.0001. MDA, malondialdehyde; T-SOD, total superoxide dismutase; GPx, glutathione peroxidase; H_2_O_2_, hydrogen peroxide.

Compared to the H_2_O_2_-model group, pretreatment with 6 μg Se/ml of SeNPs significantly alleviated IPEC-J2 cell oxidative damage caused by H_2_O_2_ (Figure 4A) and increased the living cell counts (Figure 4B). Moreover, MDA levels in the SeNPs protective group were lower than those in the H_2_O_2_ model group. T-SOD and GPx activity were higher than those in the H_2_O_2_ model group (Figure 4C). As shown in Figure 4D, the cytotoxicity dose of sodium selenite, SeMet and biogenic SeNPs on IPEC-J2 cells were 1, 32 and 64 μg/ml, respectively. Therefore, the toxicity order was as follows: sodium selenium > SeMet > biosynthesized SeNPs by *L. lactis* NZ9000.

### SeNPs Alleviates H_2_O_2_-Induced Increase of Intestinal Epithelial Permeability

**Figure 5:**
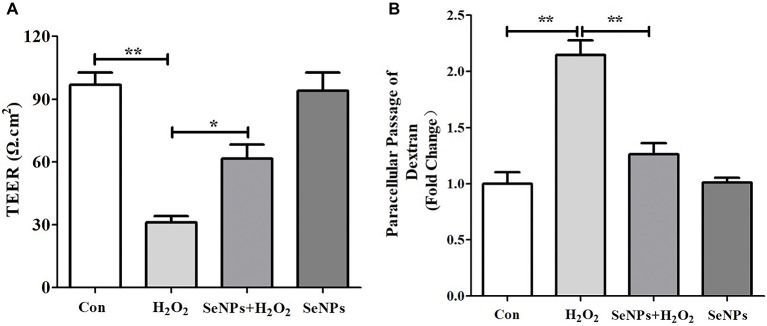
Effect of biogenic SeNPs synthesized by *L. lactis* NZ9000 on intestinal epithelial permeability. **(A)** TEER after different treatment in IPEC-J2 cells. **(B)** Effect of SeNPs on FITC-Dextran fluxes across IPEC-J2 cells exposed to H_2_O_2_. All data were presented as mean ± S.E.M. of three separate experiments. ^*^p < 0.05, ^**^p < 0.01. TEER, transepithelial electrical resistance; H_2_O_2_, hydrogen peroxide.

H_2_O_2_ remarkably caused the reduction of TEER after IPEC-J2 cells were challenged by H_2_O_2_ for 12 h, which suggests that H_2_O_2_-induced oxidative stress destroyed the intestinal epithelial integrity. However, pretreatment with SeNPs alleviated the decrease of TEER in IPEC-J2 cells exposed to H_2_O_2_ (Figure 5A). Moreover, H_2_O_2_-induced oxidative stress significantly increased the FITC-Dextran fluxes across IPEC-J2 cells compared to the control group. However, pretreatment with SeNPs obviously alleviated the increase of FITC-Dextran permeation caused by H_2_O_2_-induced oxidative stress (Figure 5B).

### SeNPs Inhibits ROS Production in IPEC-J2 Cells Exposed to H_2_O_2_

**Figure 6:**
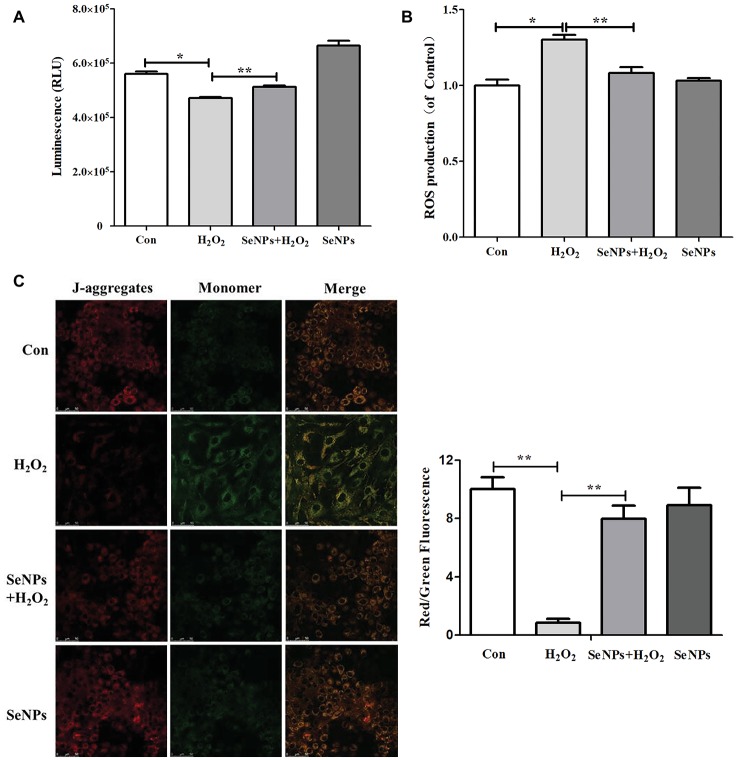
Effect of biogenic SeNPs on mitochondrial function. **(A)** The levels of ATP were measured by ATP Assay Kit. **(B)** SeNPs inhibited H_2_O_2_-induced ROS production. Intracellular ROS were detected by flow cytometry with DCFH-DA staining. **(C)** MMP were detected by MMP Assay Kit with JC-1 staining. MMP were visualized under the Confocal Microscope. All data were presented as mean ± S.E.M. of three separate experiments. ^*^p < 0.05, ^**^p < 0.01. ROS, reactive oxygen species; ATP, adenosine triphosphate; MMP, mitochondrial membrane potential.

As shown in Figure 6A, compared to the normal control group, H_2_O_2_-induced oxidative stress caused the overproduction of ROS. In contrast, SeNPs pretreatment remarkably inhibited intracellular ROS production in IPEC-J2 cells exposed to H_2_O_2_.

### SeNPs Attenuates the Decrease of Adenosine Triphosphate Caused by H_2_O_2_

Intracellular ATP levels in IPEC-J2 cells exposed to H_2_O_2_ were significantly lower than that in the normal control group. This result suggests that H_2_O_2_-induced oxidative stress affected the mitochondrial function in IPEC-J2 cells. SeNPs pretreatment attenuated the above phenomenon (Figure 6B).

### SeNPs Reduces the Loss of Mitochondrial Membrane Potential

MMP in the H_2_O_2_-model group was significantly lower than that in the normal control group. However, SeNPs pretreatment obviously reduced the loss of MMP caused by H_2_O_2_-induced oxidative stress (Figure 6C).

### Protective Effect of SeNPs on Enterotoxigenic *Escherichia coli* K88-Inudced IPEC-J2 Injury

**Figure 7:**
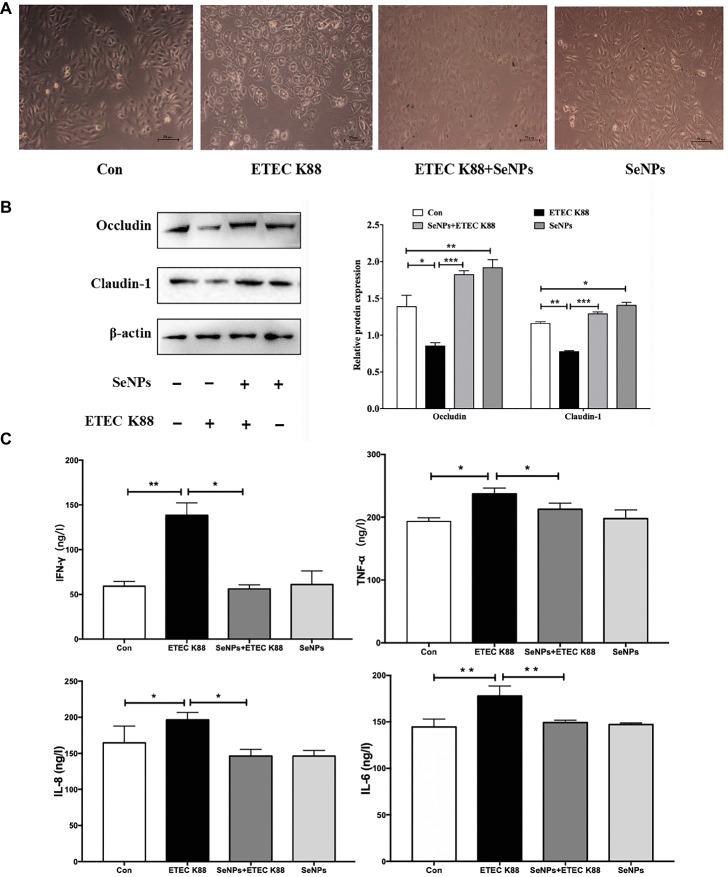
Protective effect of biogenic SeNPs on ETEC K88-induced IPEC-J2 cells injury. **(A)** Protective effect of SeNPs on IPEC-J2 cell morphology challenged by ETEC K88. **(B)** Effect of SeNPs and ETEC K88 on the expression levels of Occludin and Claudin-1. The expression levels of different tight junction proteins in IPEC-J2 from each experimental group analyzed by Western Blot. **(C)** Effect of SeNPs on the cytokines secretion in IPEC-J2 cells challenged by ETEC K88. All data were presented as mean ± S.E.M. of three separate experiments. ^*^p < 0.05,^**^p < 0.01, ^***^p < 0.001. IL-6, Interleukin 6; IL-8, Interleukin 8; IFN-γ, Interferon γ; TNF-α, Tumor necrosis factor α.

In terms of morphological change, IPEC-J2 cells treated with ETEC K88 supernatant appeared deformed, shrunken, and even caused death (Figure 7A). However, pretreatment with SeNPs significantly alleviated the toxic effect of ETEC K88 supernatant on IPEC-J2 cells. When compared to the normal control, ETEC K88 supernatant significantly down-regulated the expression levels of Occludin and Claudin-1 (Figure 7B). Conversely, pretreatment with SeNPs inhibited the above effect. In addition, pretreatment of polysaccharides-coated SeNPs significantly attenuated the increase of IL-6, IL-8, IFN-γ and TNF-α in the cell culture medium caused by the ETEC K88 challenge (Figure 7C).

## Discussion

The biological functions of Se are mainly connected with its antioxidant properties (Kielczykowska et al., 2018). Se are an essential part of selenoproteins, which contribute to antioxidant defense (Pappas et al., 2008). A hundred selenoproteins, such as GPx and thioredoxin reductase (TrxR), can be found in mammals (Broome et al., 2004; Liu et al., 2015; McKelvey et al., 2015). However, a narrow range between therapeutic and toxic doses of Se exists. Furthermore, its effects are closely related to the applied form, dose and the method of treatment. Therefore, it is a very key and complex issue to choose the most effective supplement of Se (Chakraborty et al., 2011; Han et al., 2014; Ungvari et al., 2014; Wang et al., 2014). As the attention to health effects of Se is still growing, diverse compounds of Se are still being studied including Se-enriched natural products like probiotics, yeast and green tea, as well as SeNPs (Hassanin et al., 2013; Han et al., 2014; Wang et al., 2014; Liu et al., 2015). SeNPs is a promising alternative Se supplements for applications in food, feed and biomedical fields due to its high bioavailability and low toxicity. SeNPs with a very fine particle size make it easily absorbable in the human and animal gastrointestinal tract directly, and exerts its biological function to a greater extent. Up to now, SeNPs can be synthesized by physical (Iranifam et al., 2013), chemical and biological (Cui et al., 2016) methods. As for the so-called biological technique called green synthesis, microorganisms, especially probiotic-mediated biogenic synthesis of SeNPs, possess various advantages including safety, a low-cost and high efficiency, etc. (Salunke et al., 2014; Singh et al., 2015; Cui et al., 2016). We have confirmed that probiotics *Lactobacillus casei* ATCC 393 could synthesize SeNPs. Moreover, SeNPs synthesized by *Lactobacillus casei* ATCC 393 showed lower toxicity and possess better antioxidant activity than SeMet and sodium selenite (Xu et al., 2018b). In the present study, we found that *L. lactis* NZ9000 possessed the ability to transform the toxic, colorless selenite to the non-toxic SeNPs. Moreover, biogenic SeNPs synthesized by *L. lactis* NZ9000 were mainly coated by polysaccharides and proteins, which may contribute to enhancing their stability, solubility and bioactivity. Generally, SeNPs are unstable and easily transformed into an inactive form. Biomolecules stabilized selenium nanoparticle exert scavenging activity up to 94.48% (Tareq et al., 2018). Oral administration of SeNPs-loaded chitosan/citrate complex obviously alleviated D-gal-induced oxidative stress in Kunming mice (Bai et al., 2017). Moreover, the toxicity order of different chemical forms of Se was: sodium selenite > SeMet > polysaccharides-capped SeNPs by *L. lactis* NZ9000.

Oxidative stress is a major mediator of tissue and cell injuries, and closely related to human and animal health. Selenoproteins such as GPx1 can rapidly remove H_2_O_2_ and limit the duration of H_2_O_2_ signals (Winterbourn and Hampton, 2008). As an essential part of important antioxidant enzymes such as GPx (Kielczykowska et al., 2018), Se possesses significant antioxidant activity. We found that SeNPs effectively alleviated H_2_O_2_-induced IPEC-J2 oxidative damage, which may be associated with the increase of antioxidant enzymes including GPx and T-SOD. The antioxidant effects of SeNPs are very well known (Torres et al., 2012; Zhai et al., 2017). Our previous research found that biogenic SeNPs synthesized by *L. casei* ATCC 393 possess strong antioxidant activity and can effectively protect human colon epithelial cells against H_2_O_2_ induced injury (Xu et al., 2018b).

Mitochondria are the key organelles for ATP synthesis and ROS production (Tait and Green, 2012). ROS are largely involved in causing cell damage. Mitochondrial ROS cause the increase of electron leakage and overproduction of superoxide radicals (Zoidis et al., 2018). The plasticity of the mitochondrial structure and function is a crucial property for maintaining cellular homeostasis. Previous studies indicated that an increase of paracellular permeability was observed in dinitrophenol (DNP)-induced uncouple oxidative phosphorylation of the intestinal epithelial cell model, characterized by lower TEER (Nazli et al., 2004, 2006). SeNPs functionalized with *Rosa roxburghii* polysaccharides effectively inhibited the overproduction of ROS and alle*via*ted the mitochondrial damage in INS-1 cells (Wang et al., 2019). Our obtained results indicated that biogenic SeNPs synthesized by *L. lactis* NZ9000 exhibits therapeutic potential against oxidative stress and preserves the mitochondrial function of intestinal epithelial cells, and further maintain the intestinal epithelium permeability and intestinal epithelial barrier integrity.

Various bacteria in intestine play an important role in maintaining intestinal health (Dubreuil, 2017a,b). Neonatal or early weaned animals are usually sensitive to pathogenic bacteria, which usually result in diarrhea. Besides their antioxidant properties, the antimicrobial, anticancer and immunostimulatory effects of SeNPs have been confirmed (Yazdi et al., 2013; Sonkusre et al., 2014; Cremonini et al., 2016). Se deficiency causes the increase of inflammatory factors (Piacenza et al., 2017). In order to alleviate the early weaning symptoms, SeNPs are an ideal nutritional option. ETEC strains are involved in the post-weaning diarrhea of piglets. In the study, we also observed that pretreatment with SeNPs exert a protective effect against ETEC K88-caused cytotoxicity on porcine intestinal epithelial cells, inhibit the reduction of TJ proteins, and counteracts the increase of cytokines induced by ETEC K88. Cytokines such as IL-6, IL-8, TNF-α and IFN-γ can act together to initiate and regulate the inflammation process. IL-6, as an essential cytokine, is involved in various pathologies which can be improved by IL-6 inhibition (Narazaki and Kishimoto, 2018). Nanoparticles can interact with various components of the immune system. Proinflammatory cytokines might be used to partially evaluate the immunotoxicity of nanomaterials (Elsahahy and Wooley, 2013). The current results indicate that SeNPs synthesized by *L. lactis* NZ9000 did not exhibit immunotoxicity on IPEC-J2 cells and possessed an anti-inflammatory effect on IPEC-J2 cells challenged by ETEC K88. The expression of cytokines is regulated by various mechanisms. Activation of toll-like receptors (TLRs) induces pro-inflammatory cytokines such as IL-6, TNF-α and IL-1β *via* nuclear factor-kappa B (NF-κB) (Tak and Firestein, 2001). As for the intestinal epithelium, the intercellular junctions form a selective mechanical barrier (Suzuki, 2013). Translocation of an enteric pathogen and endotoxin alters the intestinal barrier integrity by targeting intestinal epithelial TJs (Dubreuil, 2017a,b). *L. casei* ATCC 393-SeNPs protect against ETEC K88-caused intestinal barrier dysfunction (Xu et al., 2018a). The current results suggest that SeNPs maintain the intestinal epithelial barrier integrity and could be related in preventing ETEC K88-caused TJs damage, and their anti-inflammatory effects.

## Conclusions

*L. lactis* NZ9000 reduced the toxic selenite oxoanions into nontoxic elemental selenium. Isolated SeNPs was mainly coated by polysaccharides. Biogenic SeNPs protect intestinal epithelial cells against H_2_O_2_ and ETEC K88-caused injury and maintains the intestinal epithelial barrier integrity by exerting antioxidative and anti-inflammatory activities. The design of biogenic SeNPs synthesized by *L. lactis* NZ9000 offers a novel idea for the further development of a micro-ecological selenium supplement with a higher efficacy and better biosafety.

## Data Availability

All datasets generated for this study are included in the manuscript and the supplementary files.

## Author Contributions

CX and LQ were involved in the research design, execution of the experiments, analysis and interpretation of the data, drafting as well as critical revision of the manuscript. LM, YG, XD, BZ and AR provided experimental support. All authors have contributed substantially to this work and have read and approved the final manuscript for publication.

## Conflict of Interest Statement

The authors declare that the research was conducted in the absence of any commercial or financial relationships that could be construed as a potential conflict of interest.
